# RNA-seq reveals novel mechanistic targets of Livin in bladder cancer

**DOI:** 10.1186/s12894-023-01194-w

**Published:** 2023-02-28

**Authors:** Xianwen Li, Chunhua Fu, Guofeng Li, Haolin He

**Affiliations:** 1 Department of Urology, Shenzhen Yantian District People’s Hospital, 2010 Wu Tong Road, Yantian District, Shenzhen, 518081 Guangdong Province China; 2 Department of Intensive Care Medicine, Peking University Shenzhen Hospital, Shenzhen, China

**Keywords:** Bladder cancer, Livin, T24 cell, RNA-seq

## Abstract

**Background:**

Bladder cancer is a very common malignancy with a high recurrence rate. The survival of patients with muscle-invasive bladder cancer is poor, and new therapies are needed. Livin has been reported to be upregulated in bladder cancer and influence the proliferation of cancer cells.

**Materials and methods:**

The Livin gene in human bladder cancer cell line T24 was knocked out, and the differentially expressed genes were identified by RNA-seq and qPCR.

**Results:**

Livin knockdown affects gene expression and has strong negative effects on some cancer-promoting pathways. Furthermore, combined with bladder cancer clinical sample data downloaded from TCGA and GEO, 2 co-up-regulated genes and 58 co-down-regulated genes were identified and validated, which were associated with cancer proliferation and invasion.

**Conclusion:**

All these results suggest that Livin plays an important role in bladder cancer and could be a potential anticancer target in clinical therapy.

**Supplementary Information:**

The online version contains supplementary material available at 10.1186/s12894-023-01194-w.

## Introduction

Bladder cancer (BC) is the most common malignancy in the urinary system and the 13th most prevalent cause of cancer death worldwide [1]. Approximately 150,000 people die from bladder cancer each year globally [2]. There are many risk factors predispose to the disease, including genetic, anatomical, hormonal, societal, and environmental factors, as well as differential exposure to many carcinogens, such as tobacco and aromatic amines [3]. For the past 30 years, clinicians have been using limited therapeutics such as surgical resection, radiotherapy, and chemotherapy, and the 5 year survival of patients has been poor [4]. Despite many advances in the diagnosis and treatment of BC, the proportion of local recurrence and distant metastases remains high and the overall outcome is unsatisfactory. Therefore, studies addressing the BC pathogenesis and finding novel and effective biomarkers are extremely important for early diagnosis of bladder cancer and improving patient prognosis.

With the progress of medical research in recent years, targeted molecular therapies have gradually appeared in the limelight. In the 1990s, targeted molecular therapy was first proposed as a novel strategy in cancer therapy, which focuses on interfering with important cellular processes in cancer cells [5]. Many targeted agents have been investigated in BC, such as EGFR, VEGF, STAT3, and CD24, which are molecular targets that can be efficiently inhibited, resulting in a reduction of tumor growth [1, 6, 7]. Therefore, the understanding of BC biomarkers has the potential to fundamentally change the diagnosis and treatment of this disease with better clinical effects. Inhibition of apoptosis is one of the important mechanisms of cell growth in many malignant tumors. Livin (BIRC7), encoding the protein Livin, is a member of the inhibitor of apoptosis protein (IAP) family [8]. Livin consists of a single baculoviral IAP repeat domain (BIR) and a RING domain at the C-terminus that inhibits apoptosis by inhibiting proteolytic activation of capsases. Livin is also a potential anticancer target and has been found to be involved in the apoptosis through caspase-3 of human BC cells and is closely associated with recurrent BC [9]. These suggest that Livin may contribute to future targeted therapies for cancer.

In this study, we aimed to explore the role of Livin in BC, apply RNA-seq to explore the pathogenesis of BC, and then find the potential genes and their biological pathways in the BC development. This study provides a theoretical basis for novel therapeutic markers and drug treatment targets.

## Materials and methods

### Design of specific siRNAs targeting Livin

According to GenBank and the sequences of previous studies, siRNA sequences (GGACCTAAAGACAGTGCCA) were selected. siRNA-Livin-1, siRNA-Livin-2, and siRNA-Livin-3 were designed and synthesized by Guangzhou RiboBio Biotech Company (siB0711092748, Guangzhou, China).

### Cell culture and transfection

The human bladder cancer cell line T24 was purchased from Cybertron (Shanghai) Biotechnology Co., Ltd (Shanghai, China). The cell line was maintained in RPMI-1640 medium (11,875,101, Thermo Fisher, USA) with 10% FBS (10099141C, Thermo Fisher, USA), 100 U/ml penicillin and 100 μg/ml streptomycin (15,140,148, Thermo Fisher, USA). The cells were cultured at 37 °C in a standard humidified incubator with 5% CO_2_. T24 cells from the logarithmic phase were inoculated in 6-well plates containing RPMI-1640 medium. When the confluence reached 80%, the cells were assigned to different groups according to treatment: the negative control (NC) group was treated with Lipofectamine 2000 (11,668,030, Thermo Fisher, USA) only, and the si-Livin group was treated with the same volume of siRNA and Lipofectamine® 2000 reagent.

### Real-time quantitative PCR (RT-qPCR)

After 72 h of transfection, the cells were rinsed twice with phosphate-buffered saline (PBS) and then, 1 ml of TRIzol was added to lyse the cells. Total RNA was extracted according to the manual, and its quantity was measured using NanoDrop™ One (Thermo Fisher, USA). The cDNA was synthesized under conditions of 42 °C for 60 min and 72 °C for 5 min and used as a template for the amplification of the Livin gene with pre-denaturation at 95 °C for 30 s and 45 cycles of denaturation at 95 °C for 15 s, annealing and extension at 60 °C for 30 s. Real-time quantitative PCR was performed on the StepOne™ Real-Time PCR System (Life Technologies, USA). Each cDNA sample was run in triplicate, and target mRNA expression was normalized by that of GAPDH. The mRNA relative expression in each sample was calculated by using the 2^−△△CT^ analysis method. The primers were synthesized by Chi-Biotech (Shenzhen, China) and their information is shown in Table 1.

**Table 1:** The sequence information of primers

Gene name	Sequence	Length
GAPDH-F	5′-GTGAGGTGCTTCTTCTGCTATGGG-3′	259
GAPDH-R	5′-GGGCTGCGTCTTCCGGTTCTT-3′	
Livin-F	5′-AATCCCATCACCATCTTCC-3′	139
Livin-R	5′-GGACTCCACGACGTACTCA-3′	

### Western blot analysis

The cells were washed using ice-cold PBS buffer, centrifuged at 2000 rpm for 10 min, and repeated twice to collect cell precipitates. The cells were lysed by adding total cellular protein extraction reagent and then shaking in an ice bath for 30 min. After that, the cells were centrifuged at 13,000 g for 5 min at 4 °C and cellular proteins were collected. The protein concentration was detected using the BCA Protein Concentration Assay Kit. Protein samples were added with 5 × protein loading buffer, boiling at 95–100 °C for 5 min, separated by 8% SDS-PAGE, and then transferred to polyvinylidene difluoride membranes (IPVH00010, Millipore, Germany). The membranes were incubated overnight at 4 °C with the appropriate primary antibodies. The bound antibodies were then visualized using alkaline phosphatase-conjugated secondary antibodies. The intensity of the bands was quantified using the National Institutes of Health (NIH) Image program. The following antibodies were used: anti-GAPDH (1:10,000, ab181602, Abcam, UK), anti-VEGFA (1:1000, ab46154, Abcam, UK), anti-MMP-9 (1:1000, ab283575, Abcam, UK), anti-Bcl-2 (1:1000, ab196495 Abcam, UK), anti-EGFR (1:2000, #54,359, CST, USA), anti-HIF-1α (1:500, #36,169, CST, USA), anti-E-cadherin (1:1000, #3195, CST, USA), HRP-conjugated goat anti-rabbit (1:10,000, AS1107, ASPEN, China), HRP-conjugated goat anti-goat (1:10,000, AS1106, ASPEN, China), HRP-conjugated rabbit anti-goat (1:10,000, AS1108, ASPEN, China), HRP-conjugated goat anti-rat (1:10,000, AS1093, ASPEN, China), and HRP-conjugated rabbit anti-sheep (1:10,000, AS1245, ASPEN, China).

### Migration and invasion assays

Invasion experiments were performed using Transwell plates (Cat # 3422, Corning Costar). In brief, 5 × 10^4^ cells were seeded into chambers coated with Matrigel for the invasion assay in complete medium with 10% FBS. The invasion culture period was 48 h. Cells that did not penetrate the filter were wiped off, and cells on the lower surface of the filter were stained with 0.4% crystal violet. The numbers of invading cells were counted under a microscope from 5 fields in a single chamber.

### Flow cytometry analysis

Cell apoptosis was analyzed using flow cytometry. Briefly, the cell suspensions were washed with PBS and cell density was adjusted so that each well contained 5 × 10^6^ cells, and the cells were digested with trypsin and centrifuged at 1,000 rpm at room temperature for 5 min. T24 cells were stained with 100 µl of propidium iodide (PI) (P4170, Merck, Germany) and annexin V FITC (556,419, BD, USA) binding buffer and incubated for 15 min at normal temperature in the absence of light. Apoptosis was measured by a BD FACSAria III system (Beijing Jiamay Biotech) and analyzed with Modfit LT3.3 (Verity Software House, Topsham, ME, USA). For cell cycle analysis, cells were fixed and stained for 30 min with PI staining buffer containing 50 µg/mL PI and 100 µg/mL RNase A and analyzed by BD FACSAria III flow cytometer.

### RNA-seq and analysis

Different groups of T24 cells were collected, and total RNA was extracted using TRIzol. After passing the quality control by agarose gel electrophoresis, NanoPhotometer spectrophotometer (IMPLEN, CA, USA) and Bioanalyzer 2100 system (Agilent Technologies, CA, USA), the RNA samples were sent to Chi-Biotech (Shenzhen, China) for library construction, sequencing and data analysis. The libraries were sequenced using the IIIumina platform. The raw sequencing data were filtered to remove adapters and reads with low quality, and clean data were aligned to the reference genome (Human RefSeq-RNA hg19, 20,190,603) to calculate the gene expression abundance.

Differential expression analysis was performed using the R package edgeR [10, 11]. Differentially expressed genes (DEGs) were screened according to the following criteria: FDR < 0.01 and |log_2_(Fold Change)|> 1. Then, Gene Ontology (GO) enrichment analysis of differentially expressed genes was performed using the analysis software topGO [12]. Kyoto Encyclopedia of Genes and Genomes (KEGG) enrichment analysis was performed for differentially expressed genes using clusterProfiler [13] software and genes derived from corresponding pathways in the KEGG database [14], and the results were shown by bubble map.

Conventional enrichment analysis based on hypergeometric distribution relies on genes that are significantly up- or down-regulated, which tends to miss some genes that are not significantly differentially expressed but are biologically important. Therefore, Gene Set Enrichment Analysis (GSEA) analysis of GO and KEGG Pathway datasets using clusterProfiler, allows the examination of gene collections without specifying explicit differential gene thresholds.

### Acquisition and analysis of clinical transcriptome data

The RNA-seq data for 432 clinical samples were downloaded from the TCGA website (https://portal.gdc.cancer.gov/), containing 19 normal samples and 413 BC samples. And the sequences of GSE97239 with 6 clinical samples on the GEO database (https://www.ncbi.nlm.nih.gov/geo) were downloaded for mRNA expression profiling, including 3 BC clinical samples and 3 normal clinical samples. The two datasets were screened for differential genes separately, and the threshold set was FDR < 0.01 and |log2(Fold Change)|> 1. The data were analyzed jointly with the transcriptome results of the T24 cell line obtained above.

### Statistical analysis

Data were analyzed with GraphPad Prism (version 8.3). The results are expressed as mean ± the standard deviation (SD). Student’s t test was used to compare the difference between two groups, with *P* value ≤ 0.05 defined as statistically significant. All experiments were repeated three times.

## Results

### Effect of Livin knockdown in T24 cells

**Fig. 1:**
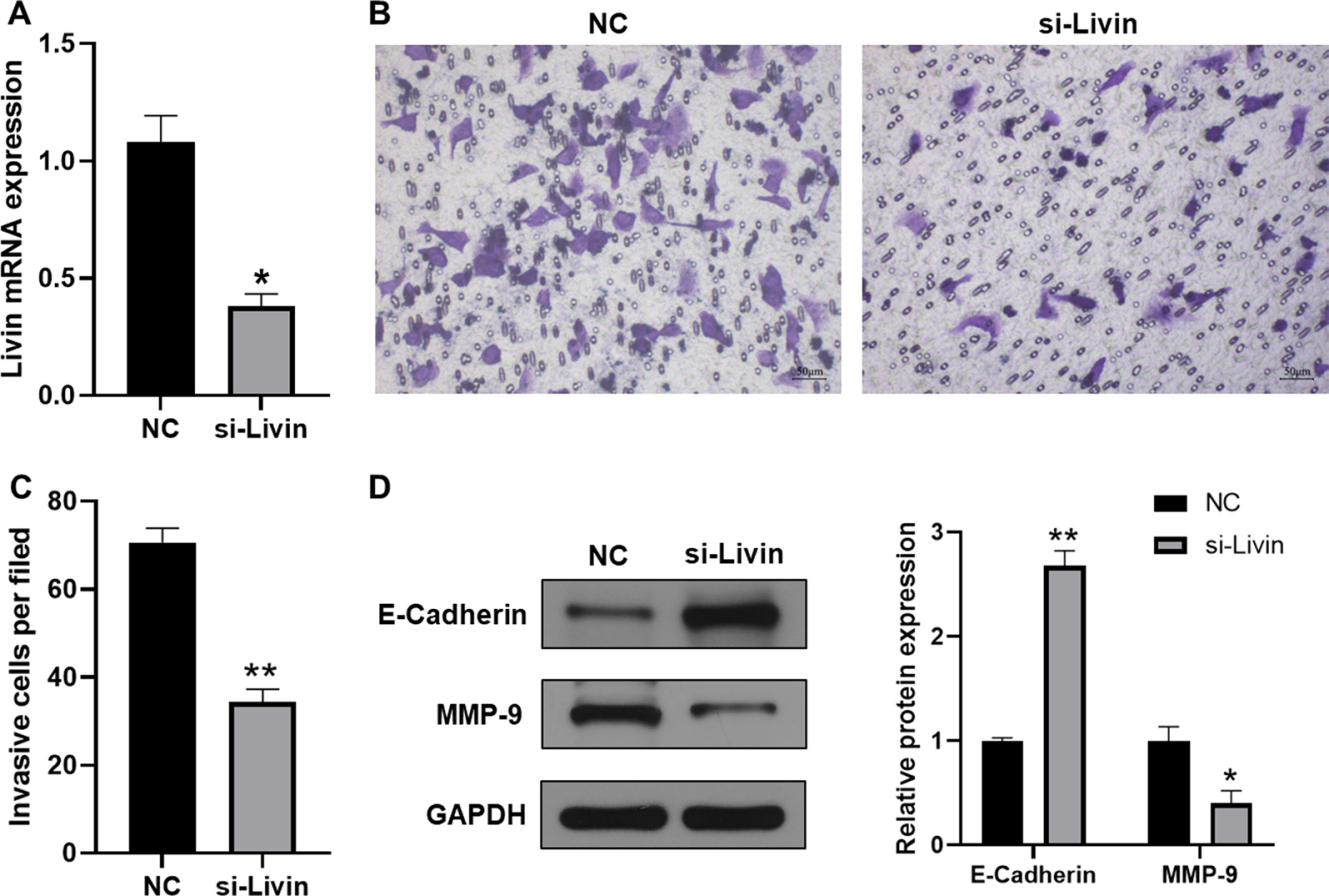
Effect of Livin knockdown in T24 cells. **A** The knockdown efficiency of Livin was detected in T24 cells. **B** Migration and invasion analysis 72 h after Livin knockdown, crystalline violet staining was performed on migrated and invading cells. **C** Efficiency of cell migration and invasion after 72 h of Livin knockdown. **D** Western blotting assays were performed to detect protein levels of E-cadherin and MMP-9 in NC group and si-Livin group, normalized by GAPDH. *P < 0.05, **P < 0.01. versus NC group

As previously described [15, 16], Livin is highly expressed in bladder cancer tissues and increases cell proliferation and survival, suggesting that Livin could be a promising marker for identifying the risk of bladder cancer recurrence. To explore the effect of Livin knockdown on cells, we investigated the effect of siRNA-mediated down-regulation of Livin on T24 cell proliferation. The human bladder cancer cell line T24 was first transfected with siRNA to achieve knockdown of the Livin gene. After 72 h of transfection, the mRNA levels of Livin expression were significantly knocked down compared to the NC group (Fig. 1A). To evaluate the effect of Livin, we assessed the relevant effect of Livin on T24 cell via cell growth assay and migration invasion analysis. Transwell invasion assay showed that si-Livin group had fewer invasive cells than NC group (Fig. 1B). Inhibition of Livin reduced the proliferation and invasion of cancer cells and increased apoptosis (Fig. 1C). Furthermore, epithelial-mesenchymal transitions (EMT) are also important processes of tumor development particularly involving in promoting tumor invasion and metastasis. Therefore, we detected the expression level of metastasis marker proteins E-cadherin and MMP-9 in the EMT process, and found that E-cadherin was upregulated in the si-Livin group, while MMP-9 was downregulated (Fig. 1D). The above results indicated that the knockdown of Livin was successful and could be used for subsequent studies.

### Effect of Livin knockdown in T24 cells on cell proliferation

**Fig. 2:**
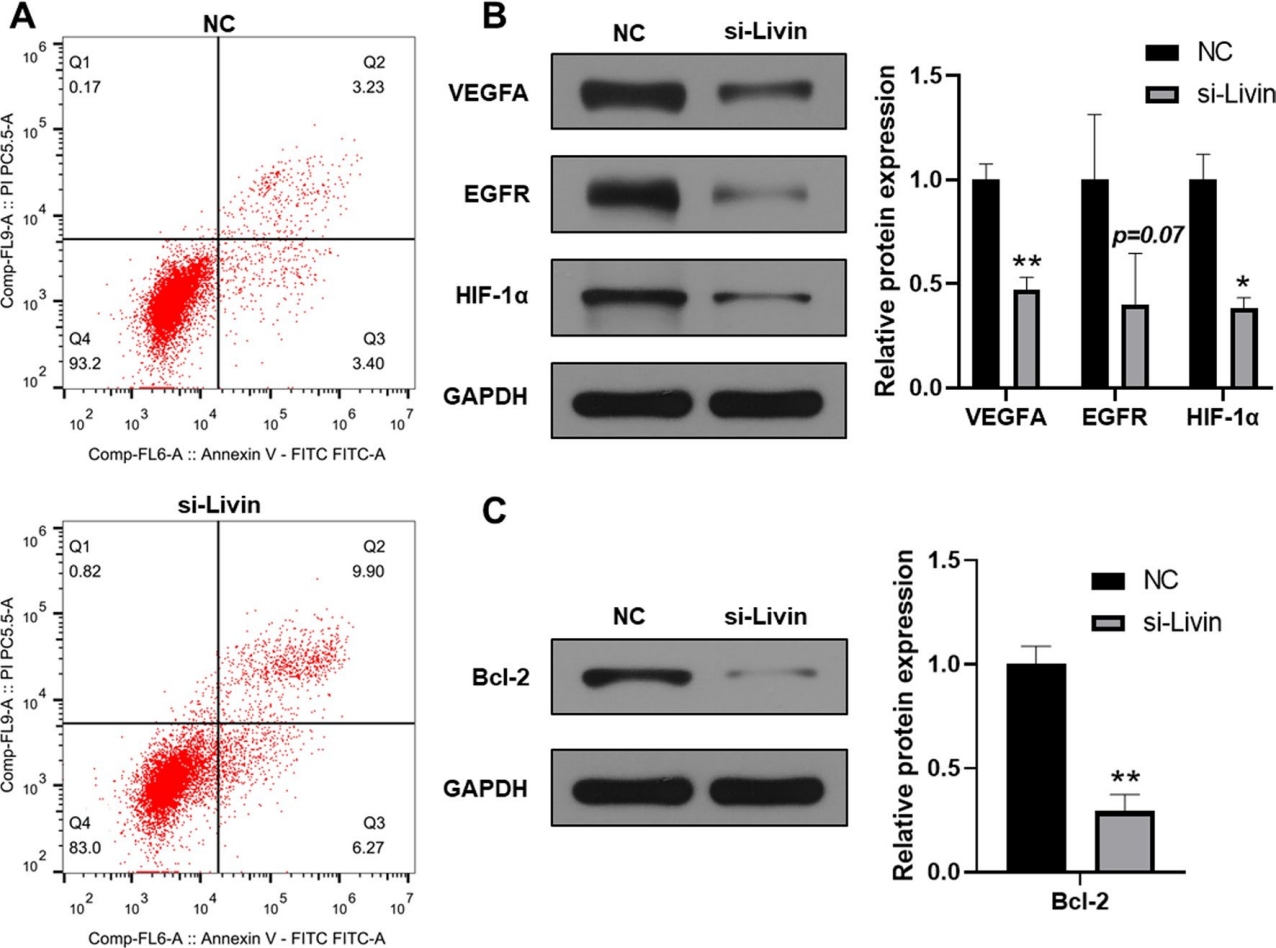
Effect of Livin knockdown in T24 cells on cell proliferation. **A** Cell apoptosis was detected by flow cytometry in NC group and si-Livin group. **B** Western blotting assays were performed to detect protein levels of VEGFA, EGFR, and HIF-1α in NC group and si-Livin group, normalized by GAPDH. **C** The protein levels of Bcl-2 in NC group and si-Livin group, normalized by GAPDH. *P < 0.05, **P < 0.01. versus NC group

To further assess the impact of Livin on cell proliferation in T24 cells, we detected a significant increase in cell apoptosis in the si-Livin group by flow cytometry analysis (Fig. 2A). Moreover, we also detected several biomarkers of cancer proliferation and cell apoptosis such as VEGFA, EGFR and HIF-α, and the anti-apoptotic factor Bcl-2. Western blot results showed that VEGFA, EGFR, HIF-α and Bcl-2 were all remarkably downregulated in the si-Livin group (Fig. 2B and C).

### Livin-regulated transcriptome was different between NC and si-Livin group

**Fig. 3:**
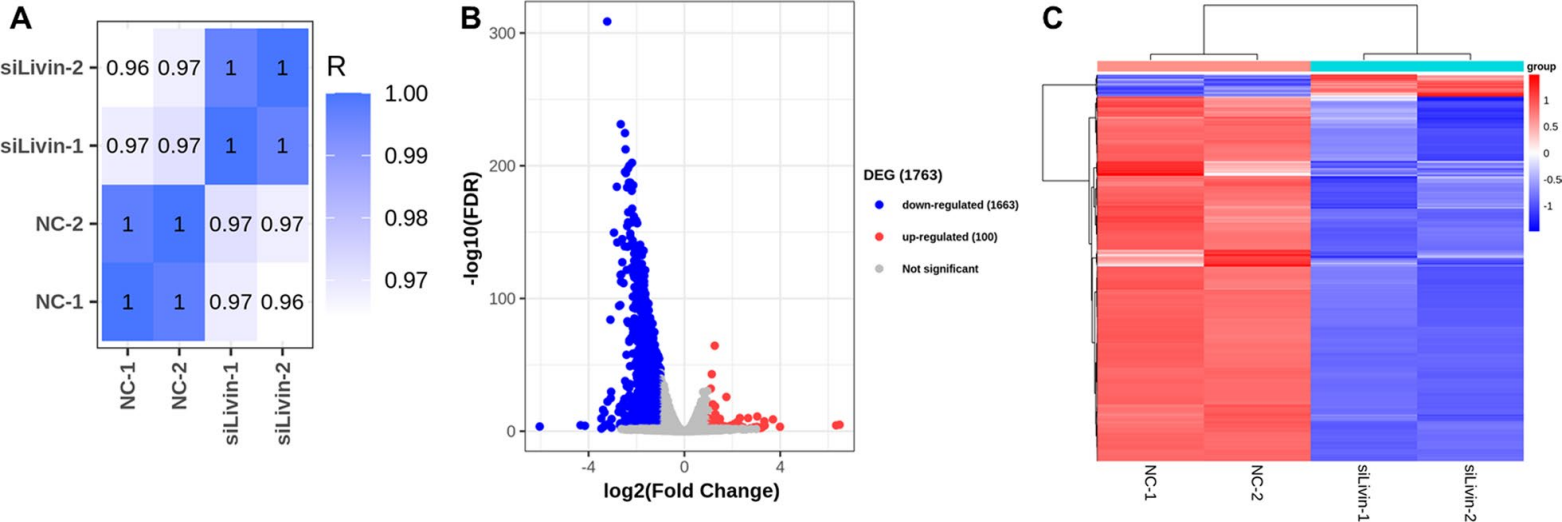
RNA-Seq revealed differences in gene expression between T24 normal cells and Livin knockdown cells. **A** The heatmap of correlation coefficients within and between sample groups. **B** Volcano diagram displayed genes upregulated and downregulated in two groups. **C** Heatmaps depicting differentially expressed genes in two groups

To detect the effect of Livin knockdown on gene expression, RNA was extracted from T24 cells in the NC and si-Livin groups for RNA-seq. The percentage of the sample sequence mapping to the reference sequence was more than 80% in both two groups (Additional file 1: Table 1). The distribution of gene expression level abundance displayed by RPKM (Reads Per Kilo bases per Million reads) is shown in Additional file 2: Fig. 1. The heat map of correlation coefficients among samples demonstrates the differences between groups and similarities within groups (Fig. 3A).

To obtain differentially expressed genes, the filtering threshold was set to FDR < 0.01 and |log_2_(Fold Change)|> 1. The volcano plot depicts the distribution of differentially expressed genes between the NC and si-Livin groups, and a total of 14,876 expressed genes were detected, of which 100 were up-regulated and 1,663 were down-regulated in si-Livin group (Fig. 3B). The heatmap demonstrates the highly consistent and si-Livin-mediated transcriptional changes in cellular gene expression for each sample (Fig. 3C).

### Enrichment analysis of DEGs biological functions

**Fig. 4:**
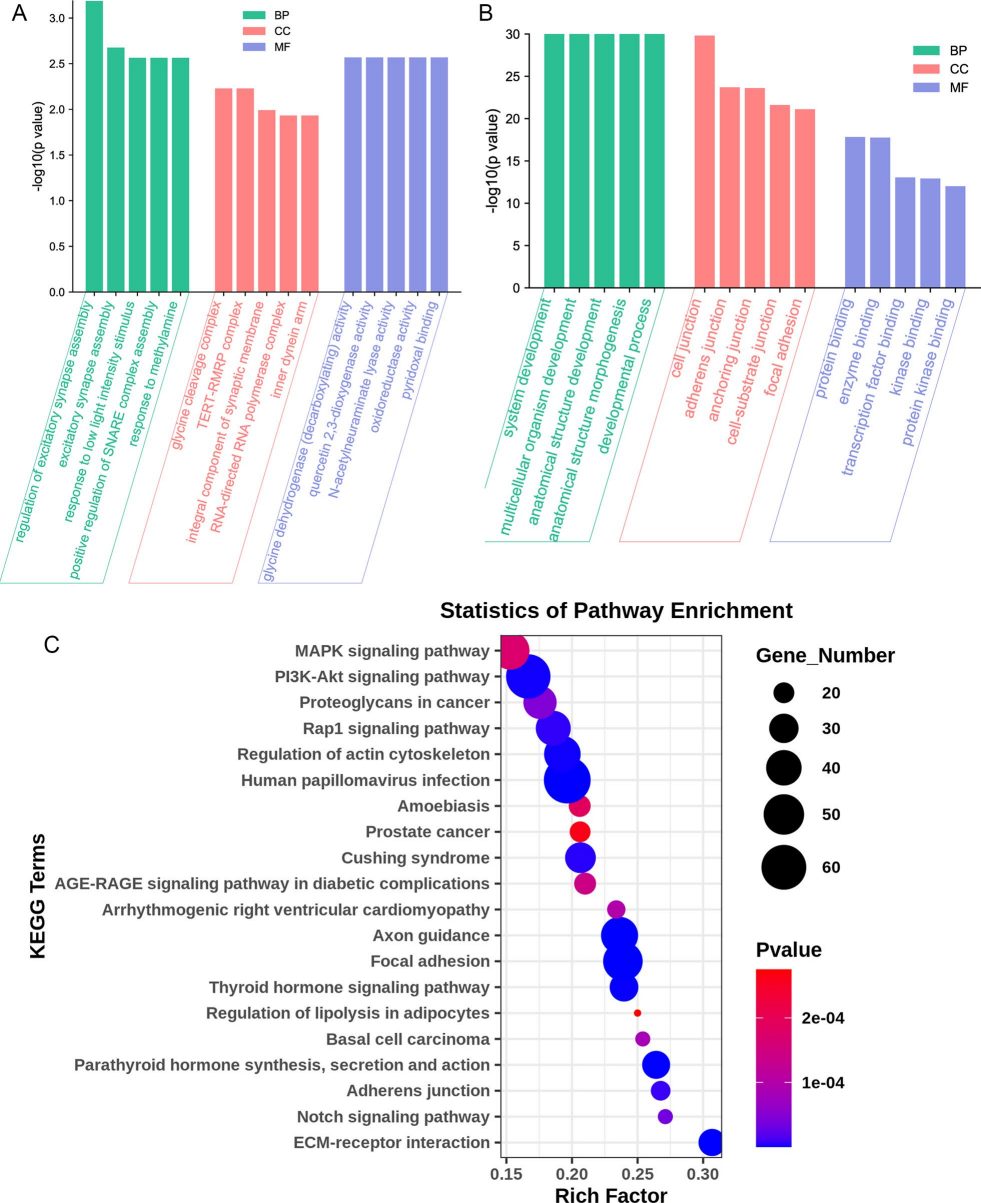
Functional analyses of the differentially expressed genes between T24 normal cells and Livin knockdown cells. **A** GO enrichment analysis for expression of upregulated genes. BP: biological process, CC: Cellular component, MF: molecular function. **B** GO enrichment analysis for expression of downregulated genes. **C** Bubble diagram of KEGG enrichment analysis results of differential genes

With the aim of characterizing the functions of genes affected by si-livin at different biological levels, GO enrichment and KEGG enrichment analysis were performed on DEGs. The GO enrichment consisted of three parts: biological processes, cellular component and molecular function. si-livin treatment resulted in the upregulation of genes associated with regulation of cell adhesion, cell proliferation, biosynthetic process, hormone catabolic process, and so on (Fig. 4A). The downregulated genes following si-livin treatment were mainly involved in regulation of signaling, cell differentiation, cell morphogenesis, and regulation of cellular process (Fig. 4B). Besides, the KEGG enrichment analysis revealed changes in gene expression associated with carcinogenesis, including ECM-receptor interaction, Notch signaling pathway, basal cell carcinoma and MAPK signaling pathway (Fig. 4C). The above biological processes and signaling pathways were highly correlated with the carcinogenic process.

### Enrichment pathways in GSEA analysis

**Fig. 5:**
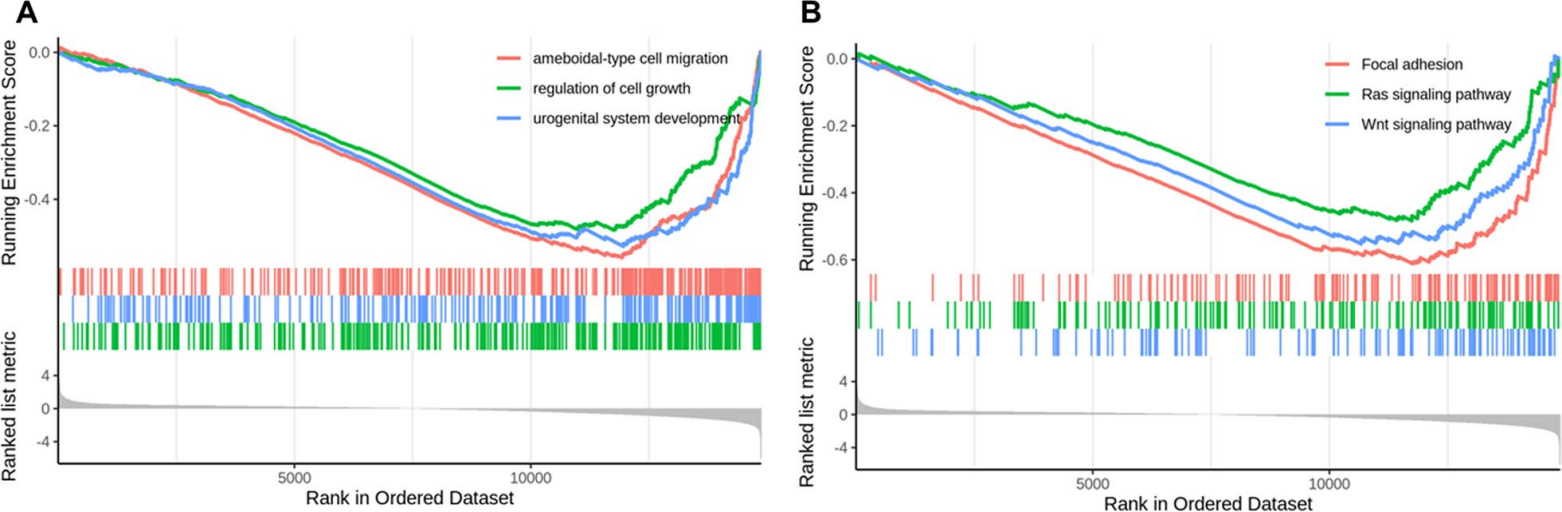
GSEA functional enrichment analysis. **A** GO enrichment analysis. **B** KEGG enrichment analysis

To further illustrate the biological pathways affected by Livin knockdown, GSEA analysis was performed on the gene sets between the NC group and the si-Livin group. GO enrichment results revealed that regulation of cell growth, urogenital system development, and ameboidal-type cell migration were negatively correlated with Livin knockdown (Fig. 5A). KEGG pathways showed that Wnt signaling, Ras signaling, and focal adhesion were negatively correlated with Livin knockdown (Fig. 5B). Overall, functional enrichment analysis identified that knockdown of Livin is associated with the regulation of cell proliferation, migration, and adhesion, which are closely related to the development of cancer.

### Screening and validation of shared differentially expressed genes by combining 2 clinical datasets

**Fig. 6:**
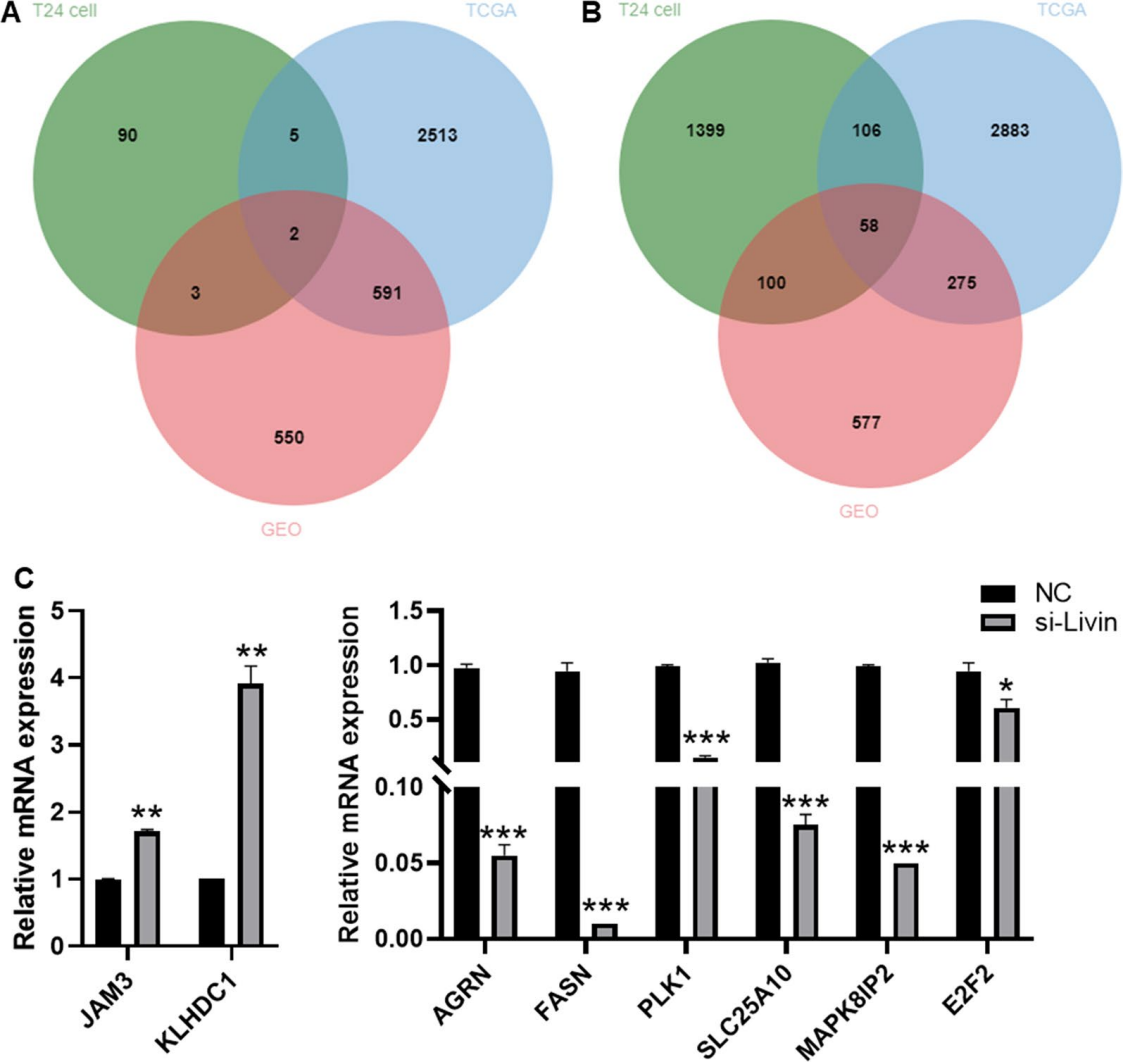
Identification of co-differentially expressed genes from T24 cell data combined with TCGA and GEO clinical data. **A** Venn diagrams of the three datasets of upregulated genes. **B** Venn diagrams of the three datasets of downregulated genes. **C** RT-qPCR validation of common genes on T24 cells. *P < 0.05, **P < 0.01, ***P < 0.001. versus NC group

We downloaded transcriptome data of 432 clinical samples from TCGA and sequences of 6 clinical samples from GEO databases, and performed differential expression analysis. Due to the difference in grouping, the normal samples from the external dataset corresponded to the si-Livin group of this study, while the cancer samples corresponded to the NC group of this study. By combining with the results of T24 cells obtained above, the results revealed the consistency of gene expression trends. Based on the differential expression trend of this study, the Venn diagram analysis revealed a total of 2 upregulated expressed genes (Fig. 6A) and 58 down-regulated expressed genes (Fig. 6B) in the 3 data sets. With the results of KEGG pathway, we selected 2 upregulated genes (JAM3 and KLHDC1) and 6 downregulated genes (AGRN, FASN, PLK1, SLC25A10, MAPK8IP2 and E2F2) for RT-qPCR validation (Table 2). From the results of RT-qPCR also indicated the upregulation of JAM3 and KLHDC1, and the downregulation AGRN, FASN, PLK1, SLC25A10, MAPK8IP2 and E2F2, sufficiently elucidating the reliability of the present study data (Fig. 6C).

**Table 2:** Differential gene data from 3 datasets

Gene	T24		TCGA		GEO	
	log_2_FC	*P* value	log_2_FC	*P* value	log_2_FC	*P* value
JAM3	1.02	1.00E-03	− 3.22	5.59E-35	− 1.97	1.24E-03
KLHDC1	1.13	2.41E-03	− 1.50	1.66E-15	− 0.52	3.16E-01
AGRN	− 2.32	3.62E-191	1.55	4.87E-11	2.66	3.02E-05
FASN	− 1.86	4.57E-129	1.45	1.24E-08	3.35	6.78E-08
PLK1	− 1.36	2.51E-69	2.27	9.53E-14	1.96	1.14E-03
TYMP	− 1.13	5.58E-29	1.73	1.47E-04	2.22	3.41E-03
COL7A1	− 1.20	3.76E-28	2.39	3.62E-07	2.36	4.94E-03
SLC25A10	− 1.14	1.60E-27	1.20	5.43E-05	1.91	1.57E-03
MAPK8IP2	− 1.54	1.11E-23	2.24	1.39E-05	2.42	3.25E-04
E2F2	− 1.27	4.74E-14	1.66	2.94E-06	1.43	9.62E-03

## Discussion

Bladder cancer is the most common malignant tumor of the urinary system and results in a major burden, with over 430,000 people diagnosed worldwide each year [17]. Due to chemotherapeutic resistance, the recurrence rate still remains high [18]. Therefore, new targets are critical for bladder carcinoma therapy. Several studies have identified potential novel targets for bladder cancer therapy in different signaling pathways. EGFR is overexpressed in many bladder tumors. Previous studies have reported that the inhibition of FGFR3 caused growth inhibition and reduced the proliferation of cancer cells [19–21]. Cetuximab, an agent used to treat advanced colorectal cancer, was used to study the effect of inhibiting EGFR in bladder cancer [22]. Nevertheless, treatment targeting FGFR3 has yielded unexpected negative results in clinical trials.

Growing evidences suggest that Livin is a specific factor that plays a role in cancer. Previous studies have indicated that Livin was expressed at low levels in most differentiated terminal tissues of adults, but was overexpressed in multiple malignant tumors [23, 24]. Wang et al. reported that Livin expression was a critical risk factor in the development and prognosis of bladder cancer, with high expression of Livin being associated with decreased patient survival [25]. In cell culture and animal models, inhibition of a target by siRNAs usually provides the first insight into target selection. In our study, we inhibited Livin with siRNA and observed a positive phenotype in T24 cells. After knockdown of Livin, some important pathways, such as the Wnt pathway, Ras pathway and focal adhesion pathway, were activated or deactivated. The abovementioned pathways are highly involved in cancer development. Wnt signaling and Ras signaling are abnormally activated in many types of cancer [19], and focal adhesion plays an essential role in tumor invasiveness and metastasis [20].

The transcriptome data of T24 cells with knockdown of Livin gene were combined with clinical data from TCGA and GEO, and the analysis identified 2 upregulated and 58 downregulated expressed genes shared by all three. JAM3, an adhesion and transmigration regulatory element, is a novel tumor suppressor in colorectal cancer, and siRNA-mediated depletion of JAM3 increased cell invasion and migration [26]. KLHDC1 knockdown reduces endoplasmic reticulum stress-induced cell death, which is essential for maintaining reactive oxygen species levels and preventing cancer development [27]. AGRN is involved in the proliferation, migration and invasion of liver cancer cells by regulating focal adhesion integrity [28], and its expression is upregulated in cancers such as hepatocellular carcinoma, promoting EMT in primary tumors [29]. The tumor-promoting function of AGRN was also demonstrated in prostate cancer [30]. Fatty acid synthase (FASN) enzyme, an androgen-regulated gene contributed to promote tumorigenesis in mice when FASN was overexpressed [31]. Genetic ablation and pharmacological inhibition of FASN in prostate cancer cells significantly inhibited cell motility and invasion [32]. PLK1 is a key regulator of the cell cycle and plays an important role in cancer development, progression and drug resistance. It was reported to be highly expressed in most human cancers (e.g., thyroid, colorectal, prostate, ovarian, etc.), and is strongly associated with poor cancer prognosis [33].SLC25A10 is upregulated in a variety of tumors and is involved in regulating intracellular levels of reactive oxygen species. Knockdown of SLC25A10 in non-small cell lung cancer cells alters growth characteristics to a less malignant phenotype and results in increased sensitivity to oxidative stress [34]. Increased expression of MAPK8IP2, a scaffolding protein that regulates the MAPK signal cascade, is significantly associated with prostate tumor progression, lymph node invasion and worse overall survival outcomes and progression-free intervals [35]. E2F2 is essential for B-Myb-induced malignant phenotype, and show positively correlation with B-Myb, interacting in colorectal cancer cells to activate ERK and AKT signaling pathways to promote carcinogenesis [36]. In gastric cancer, reducing the expression level of E2F2 inhibited the proliferation and migration of gastric cancer cells [37]. All of these genes have been reported to be involved in cancer development, migration, and invasion, and in-depth studies of these genes have the potential to discover novel bladder cancer target molecules.

In conclusion, knockdown of Livin inhibited cell proliferation and motility and increased cell apoptosis. RNA-seq results showed that knockdown of Livin resulted in the differential expression of many cancer-related genes and influenced many pathways correlated with cancer. This study provides evidence to further delineate the role of Livin in bladder cancer and the underlying mechanism.

## Supplementary Information

**Additional file 1: Table S1.** Statistics of sequencing data.

**Additional file 2: Fig. S1.** The distribution of gene expression level abundance displayed by RPKM. **A** The diagram of RPKM distribution. **B** The curve of RPKM density distribution.

## Data Availability

The datasets generated and/or analysed during the current study are available in the Zendo repository, Accession number [7192146].
