# Supplementary material for: RNA-seq reveals novel mechanistic targets of Livin in bladder cancer
Source: BMC Urol. 2023 Feb 28;23:26. doi: 10.1186/s12894-023-01194-w (PMC9976429; doi:10.1186/s12894-023-01194-w)
Supplement: Supplementary file 1 — Additional file 1: Table S1. Statistics of sequencing data. [file 12894_2023_1194_MOESM1_ESM.docx]

| **Supplementary Table 1. Statistics of sequencing data.** | | | | |
| --- | --- | --- | --- | --- |
| Terms | NC-1 | NC-2 | si-Livin-1 | si-Livin-2 |
| Raw Reads | 23,544,701 | 26,107,172 | 22,650,920 | 23,388,221 |
| Clean Reads | 23,229,669 | 25,680,786 | 20,769,729 | 21,752,206 |
| Q20 | 96.98% | 96.66% | 96.75% | 96.51% |
| Q30 | 92.10% | 91.47% | 91.66% | 91.17% |
| GC% | 50.83% | 50.53% | 49.99% | 48.88% |
| Mapped Reads | 19,540,373 | 21,522,256 | 16,824,378 | 17,582,661 |
| Mapping Rate | 84.12% | 83.81% | 81.00% | 80.83% |
