# Supplementary material for: RNA-seq reveals novel mechanistic targets of Livin in bladder cancer
Source: BMC Urol. 2023 Feb 28;23:26. doi: 10.1186/s12894-023-01194-w (PMC9976429; doi:10.1186/s12894-023-01194-w)

**Supplementary Figure 1. The distribution of gene expression level abundance displayed by RPKM. (A)** The diagram of RPKM distribution. **(B)** The curve of RPKM density distribution.


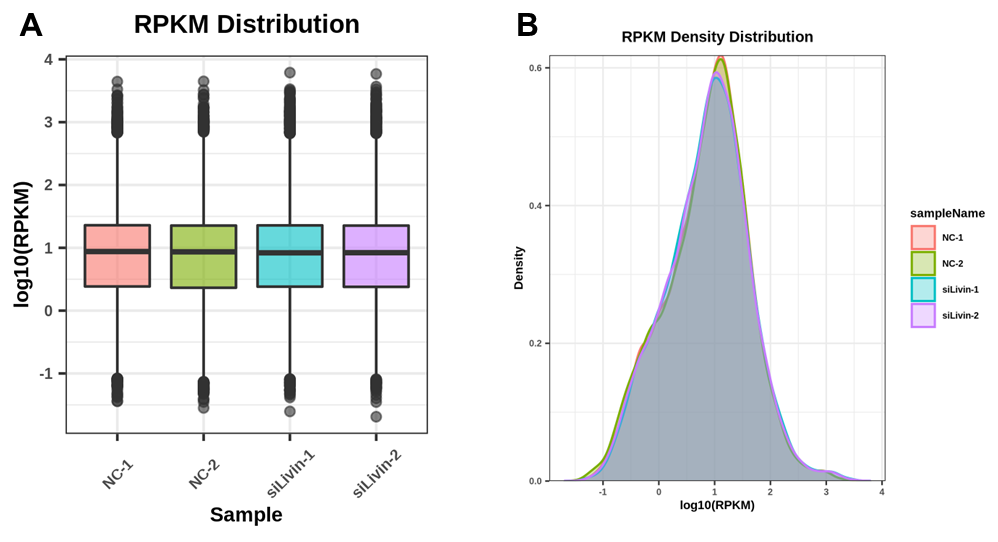

Supplement: Supplementary file 2 — Additional file 2: Fig. S1. The distribution of gene expression level abundance displayed by RPKM. A The diagram of RPKM distribution. B The curve of RPKM density distribution. [file 12894_2023_1194_MOESM2_ESM.docx]
